# Ion Channel Disorders and Sudden Cardiac Death

**DOI:** 10.3390/ijms19030692

**Published:** 2018-02-28

**Authors:** Anna Garcia-Elias, Begoña Benito

**Affiliations:** 1 Group of Biomedical Research in Heart Diseases, Hospital del Mar Medical Research Institute (IMIM), C/Doctor Aiguader 88, 08003 Barcelona, Spain; agarciaelias@imim.es; 2 Cardiology Department, Hospital del Mar, Passeig Marítim 25-29, 08003 Barcelona, Spain

**Keywords:** sudden cardiac death, channelopathies, ion channel, primary electrical disorders, long QT syndrome, short QT syndrome, Brugada syndrome, catecholaminergic polymorphic ventricular tachycardia

## Abstract

Long QT syndrome, short QT syndrome, Brugada syndrome and catecholaminergic polymorphic ventricular tachycardia are inherited primary electrical disorders that predispose to sudden cardiac death in the absence of structural heart disease. Also known as cardiac channelopathies, primary electrical disorders respond to mutations in genes encoding cardiac ion channels and/or their regulatory proteins, which result in modifications in the cardiac action potential or in the intracellular calcium handling that lead to electrical instability and life-threatening ventricular arrhythmias. These disorders may have low penetrance and expressivity, making clinical diagnosis often challenging. However, because sudden cardiac death might be the first presenting symptom of the disease, early diagnosis becomes essential. Genetic testing might be helpful in this regard, providing a definite diagnosis in some patients. Yet important limitations still exist, with a significant proportion of patients remaining with no causative mutation identifiable after genetic testing. This review aims to provide the latest knowledge on the genetic basis of cardiac channelopathies and discuss the role of the affected proteins in the pathophysiology of each one of these diseases.

## 1. Introduction

Sudden cardiac death (SCD) is an unexpected death defined as that occurring within the first hour of the onset of symptoms [1]. It is responsible for 250,000–300,000 deaths annually in the US, with an estimated annual incidence of 50–100/100,000 individuals in industrialized countries [2]. It is therefore a major public health problem, with a tremendous social impact that becomes particularly devastating when affecting young people [3]. Ventricular arrhythmias, especially ventricular fibrillation, are the major underlying rhythms that lead to SCD. In individuals over 35, SCD most commonly occurs in patients with coronary heart disease with or without heart failure, whereas inherited cardiomyopathies and primary electrical disorders prevail in younger SCD victims [4]. Hereditary primary electrical disorders may account for up to 30% of all SCD in the young [5], and primarily include the long QT syndrome (LQTS), the short QT syndrome (SQTS), the Brugada syndrome (BrS) and the catecholaminergic polymorphic ventricular tachycardia (CPVT). These disorders, also known as cardiac channelopathies, most commonly respond to a mutation(s) in a gene(s) encoding cardiac ion channels or receptors and/or their regulatory proteins, the consequence in all cases being a modification in the cardiac action potential or in the intracellular calcium handling that leads to electrical instability and predisposition to life-threatening ventricular arrhythmias [6]. Recently, a disorder termed “early repolarization syndrome” has emerged as a new potential channelopathy. Although defined by a characteristic electrocardiogram (ECG) pattern that has been shown to be more common among patients experiencing SCD [7], most of the potential genetic variants associated with the disorder lack functional or biological validation, so it is still an unresolved issue whether this is a true primary electrical disorder [8]. For this reason, this review will leave out this syndrome and focus on the four above-mentioned channelopathies.

Considering that cardiac channelopathies supervene in patients without structural heart disease that are usually unaware of their disorder and that SCD might be the first presenting symptom, early diagnosis of genetic carriers is warranted. With very few exceptions, cardiac channelopathies are autosomal dominant disorders with incomplete penetrance (meaning that some individuals will not express the trait even though they carry the mutated allele) and variable expressivity (meaning that the level of phenotypic expression will be diverse for individuals with the same genotype) [9]. This represents a significant limitation when the diagnosis is based exclusively on clinical findings. Genetic testing may (1) offer a definite confirmation of a particular electrical disorder, which becomes particularly useful in patients with inconclusive clinical data; (2) confirm or exclude the presence of a disease-causing mutation in family members of an index case; and (3) help personalize treatment recommendations and management of a patient’s specific disorder [9]. Therefore, in recent years genetic testing has been progressively introduced in clinical practice and is currently advised (with different levels of recommendation) in most cardiac channelopathies [10]. However, genetic screening of mutations in genes known to cause cardiac channelopathies might result unsuccessful, providing negative results in around 20% of patients with LQTS, 40% of patients with CPVT, and 80% of patients with SQTS or BrS [9]. This indicates that there is still a long way to go in the field of genetics in primary electrical disorders. This review aims to provide the latest knowledge on the genetic basis of cardiac channelopathies and discuss the role of the affected proteins in the pathophysiology of each one of these diseases. Of note, because of the ongoing description of novel mutations associated with these disorders, and as suggested elsewhere [11], for the sake of simplicity, we will avoid naming these entities by a numerical subtype, except for the major susceptibility genes (i.e., LQT1, LQT2, and LQT3; SQT1; BrS1; and CPVT1).

## 2. Cardiac Electrical Physiology: Role of Ion Channels and Receptors

**Figure 1:**
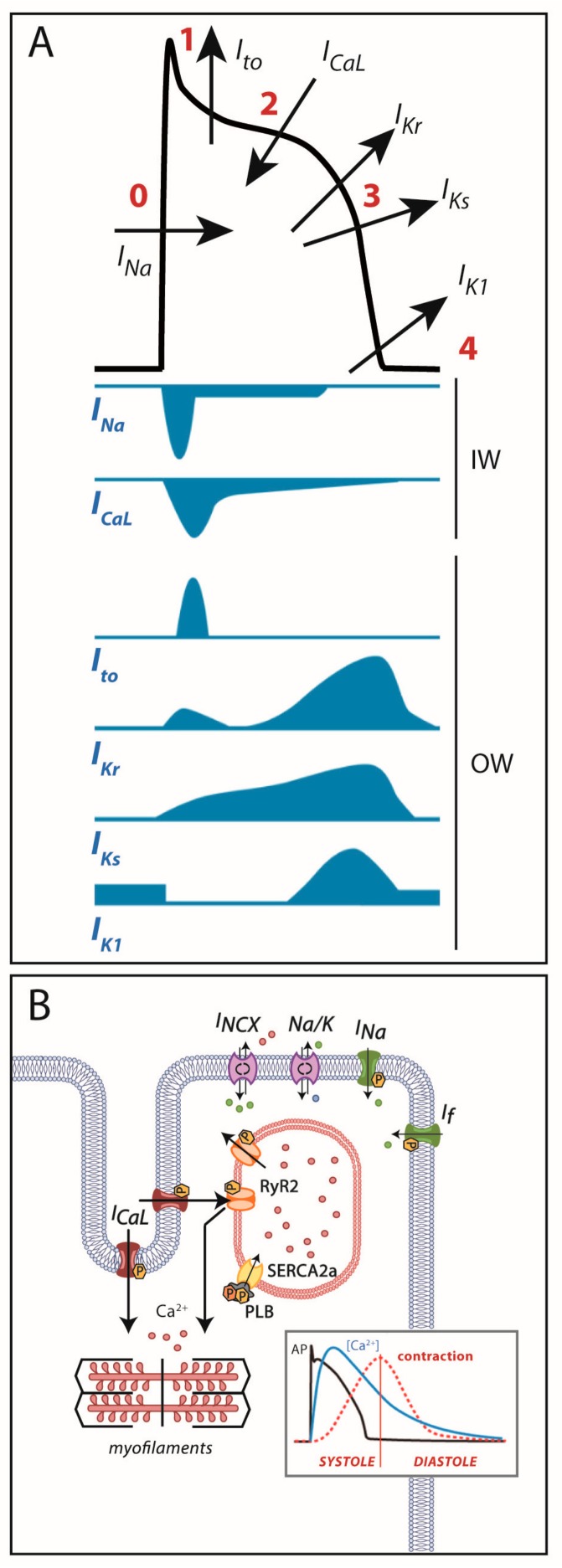
(**A**) Cardiac action potential and transmembrane ionic currents that participate in each phase. Phase 0: rapid depolarization due to entrance of Na^+^ currents into the cell. Phase 1: early repolarization initiated by outward K^+^ *I_to_* currents. Phase 2: a plateau phase marked by the Ca^2+^ entry into the cell against K^+^ outward repolarizing currents. Phase 3: end of repolarization produced by K^+^ currents upon Ca^2+^ channel-inactivation. Phase 4: resting membrane potential (≈−90 mV) determined by inward-rectifier K^+^ currents. OW: outward currents. IW: inward currents. (**B**) Excitation-contraction coupling: during action potential, Ca^2+^ entry in phase 2 induces a large release of Ca^2+^ from the sarcoplasmic reticulum through the RyR2 receptor that allows cell contraction. After repolarization, Ca^2+^ is extruded from the cell through the Na^+^/Ca^2+^ exchanger or taken back into the sarcoplasmic reticulum through SERCA2a to allow cell relaxation.

Cardiac cells are excitable cells with the ability of generating and propagating an action potential (AP), an electrical signal that will translate into cardiomyocyte contraction. The cardiac AP is generated by a set of ion movements across the cell membrane that take the cell from the resting state to an activated state (by depolarization) and back to the resting membrane potential (repolarization). All phases are the consequence of a synergistic activation and inactivation of several voltage-dependent ion channels. In contractile myocytes (Figure 1A), APs are triggered by the acute entrance of sodium ions (Na^+^) inside the cell, resulting in an inward current (*I_Na_*) that shifts the membrane potential from its resting state (−90 mV) to a depolarization state (+20 mV). This phase is followed by the efflux of potassium (K^+^) ions through an outward current named *I_to_*, which initiates cell repolarization. Consecutively comes the plateau phase, a short period of constant membrane potential due to the balance between inward calcium (Ca^2+^) currents (*I_CaL_*) through the L-type voltage-dependent calcium channels (LTCC) and time-dependent delayed-rectifier outward K^+^ currents (mainly slow delayed-rectifier *I_Ks_* and rapid delayed-rectifier *I_Kr_*). At this point, the Ca^2+^ entry through LTCC triggers a much larger release of Ca^2+^ from the sarcoplasmic reticulum (SR) stores through the ryanodine receptor channel type 2 (RyR2), producing a systolic increase in intracellular Ca^2+^ needed for cell contraction (Figure 1B). Upon inactivation of the LTCC, the net outward K^+^ currents repolarize the cell and bring the membrane potential to its resting state. The balance between Ca^2+^ and K^+^ currents, therefore, determines the AP duration. The basal and acetylcholine-dependent inward-rectifier K^+^ currents (*I_K1_* and *I_KACh_*) control final repolarization and determine the resting membrane potential (Figure 1A). Ca^2+^ is then extruded from the cell through the Na^+^/Ca^2+^ exchanger (NCX) type 1 and taken back into the SR through the SR Ca^2+^-ATPase type 2a (SERCA2a). This restores low intracellular Ca^2+^ levels, allowing cell relaxation during diastole (Figure 1B).

As shown in Figure 1, all these processes are tightly mediated by multiple ion channels and regulatory proteins. The slightest modification in the functioning of one of these players may modify the AP and/or the intracellular Ca^2+^ dynamics, potentially favoring an arrhythmogenic substrate. In the following lines we will discuss how the electrical substrate of cardiac channelopathies can develop depending on the affected gene and the effect of a particular mutation (loss- or gain-of-function) on the channels, receptors and regulators that participate in this process.

## 3. Long QT Syndrome

**Figure 2:**
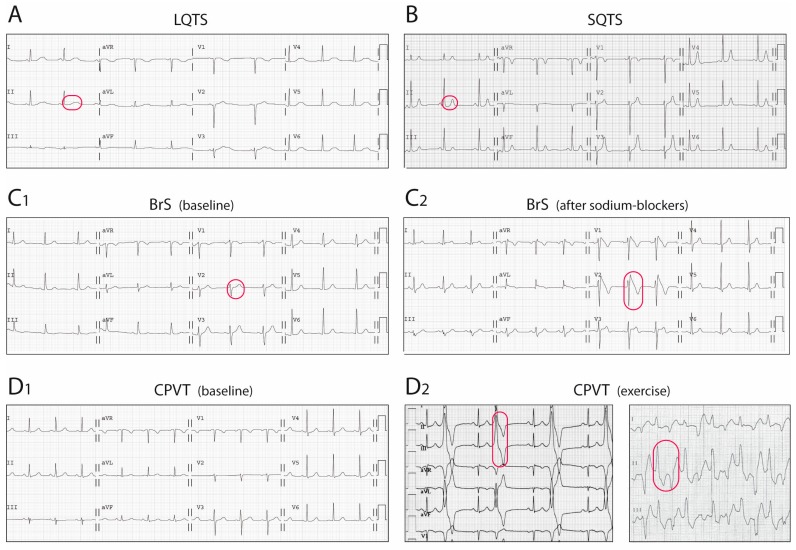
Electrocardiographic findings in the four primary electrical disorders or channelopathies. (**A**) LQTS, with prolonged QT interval; (**B**) SQTS, with shortened QT interval; (**C**) BrS. In this case, ECG at baseline was normal (C1), but the typical pattern, with ST-segment elevation in right precordial leads, was unmasked after a provocative test with sodium-blockers (D2); (**D**) CPVT. ECG is normal at baseline (D1), but premature ventricular complexes and occurrence of bidirectional tachycardia appear with exercise (D2). Characteristic ECG features of each disorder are circled in red.

**Figure 3:**
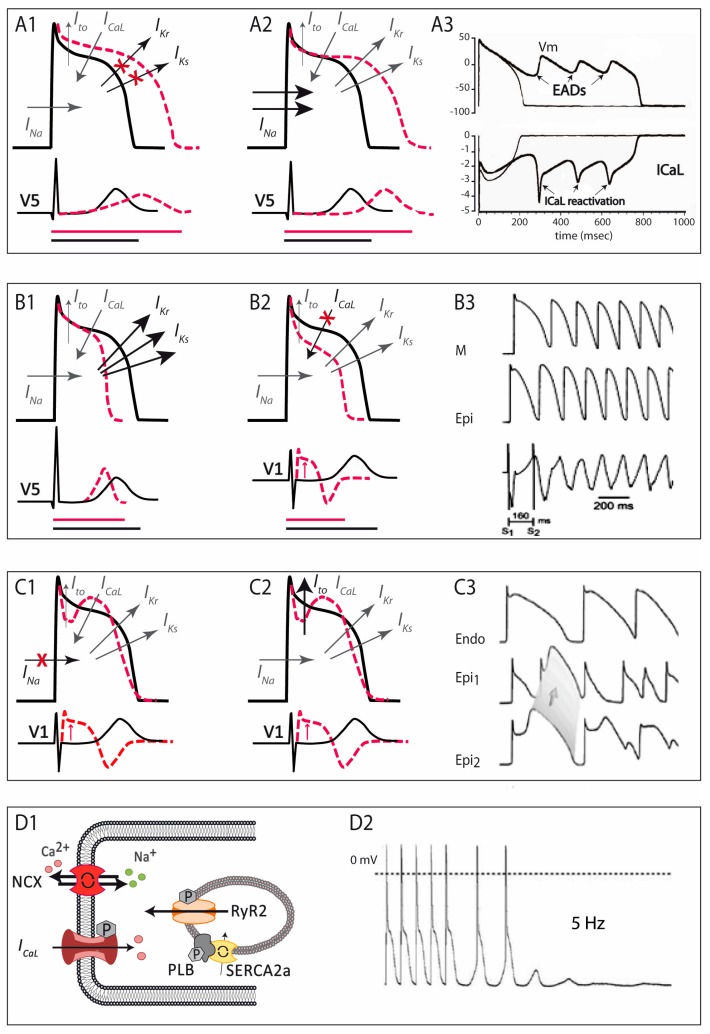
Schematic representation of the pathophysiological mechanisms involved in the four main primary electrical disorders. (**A**) in LQTS, either by a decrease in K^+^ currents (A1) or an increase in Na^+^ currents (A2), AP duration is prolonged, and so is the QTc interval on the ECG (depicted by the bottom horizontal lines: in black normal QT, in red long-QT interval). This situation favors the development of early afterdepolarizations, the trigger of ventricular arrhythmias in LQTS patients (A3). (**B**) in SQTS, an increase in K^+^ currents accelerates repolarization (B1), and manifests as short QTc interval in the ECG (depicted by the bottom horizontal lines: in black normal QT, in red short-QT interval); in those cases of SQTS caused by loss-of-function mutations in the calcium channel (B2), besides shortening of the AP duration there is transmural gradient in early phases of repolarization, leading to ST-segment elevation like the one seen in BrS (combined phenotype, QT interval depicted by horizontal lines and ST segment elevation by the red arrow). In SQTS, an increased dispersion of repolarization favors the appearance of atrial and ventricular arrhythmias (B3). (**C**) In BrS, a decrease in Na^+^ currents (C1) or, less commonly, an increase in *I_to_* currents (C2); produces a ionic imbalance in early repolarization, giving rise to the characteristic ST-segment elevation seen in the ECG (depicted by the red arrows). The consequent epicardial and transmural dispersion of repolarization favors ventricular arrhythmias by a mechanism of phase-2 reentry (C3). (**D**) CPVT is produced by abnormal Ca^2+^ leak from the sarcoplasmic reticulum (D1), which favors the occurrence of delayed afterdepolarizations, which in turn can trigger ventricular arrhythmias (D2). Modified from [6].

The LQTS is a hereditary electrical disorder characterized by an abnormally prolonged QT interval on the ECG (Figure 2A), which reflects a prolonged repolarization in cardiac cells secondary to changes in ion currents participating in the AP (see below). In LQTS, prolonged repolarization allows time for LTCC to recover from inactivation and facilitate new Ca^2+^ entry that can potentially generate a new depolarization (early afterdepolarization), a mechanism known to promote arrhythmogenesis (Figure 3A) [6]. As such, LQTS patients may present ventricular arrhythmias such as torsades de pointes and SCD.

### 3.1. Clinical Features

The estimated prevalence of the LQTS is thought to be as high as 1/2,000 individuals [12]. The diagnosis of LQTS is currently established in [13]: (1) the presence of a QT interval corrected for heart rate (QTc) ≥ 480 ms in repeated 12-lead ECGs; (2) the presence of a confirmed pathogenic LQTS mutation, irrespective to the QT duration; (3) a LQTS risk score, based on symptoms, family history and ECG findings [14], >3 in the absence of a secondary cause for QT prolongation.

Although most patients remain asymptomatic throughout life, up to 13% may experience SCD and 36% may experience syncope before age 40 if untreated [15]. SCD and syncope are the clinical manifestations of sustained and non-sustained ventricular arrhythmias, respectively. The mean age at the time of the first symptom is around 14 [13]. Symptoms in LQTS are characteristically triggered by different circumstances that emphasize the underlying electrical disorder (see below), such as exercise (in LQT1), emotions (in LQT2) or sleep (in LQT3) [16]. Several factors have been shown to be related to the occurrence of arrhythmias in LQTS patients, and therefore must be actively assessed in clinical practice: the presence of prior SCD or syncope, a QTc interval in any ECG longer than 500 ms despite treatment with beta-blockers, and female sex in LQT2 and LQT3 patients [13,15,17]. Certain genetic findings have also shown prognostic significance (see below).

First-line treatment of all LQTS patients at the moment of diagnosis comprises the implementation of certain lifestyle changes (like avoidance of QT-prolonging drugs and genotype-specific triggers for arrhythmias) and treatment with β-blockers [13]. Additionally, an implantable cardioverter defibrillator (ICD) is indicated in patients considered at high risk of fatal arrhythmias, such as in survivor of a previous cardiac arrest, those with syncope or ventricular tachycardias despite treatment with β-blockers, and those with LQT2 and QTc > 500 ms persistently [13]. Genetic testing, which provides conclusive results in up to 80% of patients, particularly directed to the three most prevalent LQTS-susceptible genes (*KCNQ1*, *KCNH2*, and *SCN5A*) is also recommended in all patients with diagnosis of LQTS [10].

### 3.2. Genetic Bases

As mentioned earlier, LQTS is characterized by prolonged AP duration, which can be consequence of an increase in inward currents (mainly *I_Na_* and *I_CaL_*) or a decrease in outward K^+^ currents (mainly *I_Ks_*, *I_Kr_*, *I_K1_*, Figure 3A). Mutations in 20 different genes encoding direct or indirect mediators of these currents have been found in one or several families with LQTS. Table 1 summarizes the genes, their corresponding proteins, and the effect of the LQTS-associated mutations. Most of the described pathogenic variants are inherited in an autosomal dominant pattern, with the exception of those embraced in the Jervell and Lange–Nielsen syndrome (a rare syndrome combining extremely prolonged QT interval and congenital deafness associated to mutations in *KCNQ1* and *KCNE1*) [13] and a reported family with a mutation in *TRDN* [18], with autosomal recessive inheritance pattern. Autosomal dominant forms LQTS include LQT types 1–13, of which LQT1, LQT2 and LQT3 represent 85% of all genetically-diagnosed cases [19].

LQT1, accounting for >40% of all LQTS, is caused by mutations in the *KCNQ1* gene encoding K_V_7.1, the α-subunit of the voltage-dependent K^+^ channel that mediates the slow component of the delayed rectifying *I_Ks_* current. LQT1-related mutations in this gene cause a loss-of-function of the channel and less *I_Ks_*, explaining the prolonged AP duration and the QT interval on the ECG [20,21]. Since *I_Ks_* currents respond to progressive adrenergic stimulation such as that present during exercise, LQT1 patients show a marked trend to present arrhythmia-related symptoms during sport practice [16]. The effect of different mutations over the final Kv7.1 function may have prognostic implications: mutations found in the cytoplasmic-loop transmembrane domains, involved in adrenergic channel regulation, are associated with a greater risk of arrhythmias triggered by exercise but not with those during rest or sleep [22,23].

LQT2 (representing 30% of all LQTS) is caused by mutations in the *KCNH2* gene encoding K_V_11.1 (or hERG), the α-subunit of the voltage-dependent K^+^ channel mediator of the rapid component of the delayed rectifying *I_Kr_* current. LQT2-related mutations entail a loss of function of *KCNH2*, in some instances by altering its traffic to the cell membrane, with a consequent reduction in *I_Kr_* [24,25,26]. Since *I_Kr_* plays an important role in brisk increases in heart rate, LQT2 patients are prone to present arrhythmia-related symptoms in stress or emotional circumstances [16]. Location of LQT2-related mutations may be relevant to predict a greater reduction of *I_Kr_* and thus a more aggressive phenotype: as such, patients with mutations affecting the pore region (S5-loop-S6), a determinant structure of the channel, have been reported to have higher risk of suffering life-threatening cardiac events than patients with mutations in other locations [27,28].

Finally, LQT3 is caused by mutations in *SCN5A* encoding Na_V_1.5, the α-subunit of the voltage-dependent Na^+^ channel and mediator of the depolarizing *I_Na_* current. Gain-of-function mutations in this gene, present in 10% of genetically-diagnosed LQTS patients, prolong AP duration by increasing late depolarizing currents [29]. In LQT3 patients, the defect in *I_Na_* becomes more evident with slow heart rates, so it is common that these patients develop arrhythmia-related symptoms in circumstances of bradycardia and typically during sleep [16]. Although some particular mutations (Na_V_1.5-E1784K and Na_V_1.5-D1790G, also associated with Brugada phenotype) have been related to a better prognosis in LQT3 patients, at present there is not enough evidence supporting that the type and/or location of *SCN5A* mutations in LQT3 patients have effects on the final phenotype [30].

Mutations in other genes have also been reported in LQTS patients, mainly anecdotically, representing each of them <1% of global cases. As mentioned, all of them lead to a decrease in outward currents or an increase in inward currents directly or indirectly (Table 1), the effect in all cases being a prolongation of AP duration and repolarization:

- Uncommon LQTS mutations causing a decrease in outward currents. Two genes encoding regulatory β-subunits of K^+^ channels have been associated with the LQTS: mutations in *KCNE1*, encoding minK, the β1-subunit of voltage-dependent K^+^ channels, have been reported to interfere with the traffic of K_V_7.1 and lead to a reduction of *I_Ks_* current [31,32]; on the other hand, mutations in *KCNE2*, encoding MiRP1 (minK-related peptide 1), have been reported to impair K_V_11.1 kinetics (slower activation, faster deactivation and increased drug sensitivity), inducing a decreased *I_Kr_* [33]. Among the inward-rectifying K^+^ channel family, two members have also been associated to LQTS, *KCNJ2* and *KCNJ5*. *KCNJ2* encodes Kir2.1, the inward rectifying K^+^ channel that mediates the *I_K1_* current [34]. Loss-of-function mutations in this gene lead not only to LQTS and susceptibility to arrhythmias, but also to periodic paralysis and developmental abnormalities, a condition known as the Andersen–Tawil syndrome [35]. *KCNJ5* encodes Kir3.4, the inward rectifying K^+^ channel mediator of the acetylcholine/adenosine-induced *I_K,Ach_* current. Loss-of-function mutations in this gene lead to LQTS by altering the traffic of the channel [36,37]. Finally, mutations in AKAP, a gene that encodes an auxiliary protein (*AKAP9*, A-kinase anchor protein-9) and not an ion channel, may also cause LQTS by reducing outward currents. *AKAP9* is a scaffolding protein between K_V_7.1 and PKA. Mutations in AKAP have been shown to reduce the K_V_7.1/PKA interaction, resulting in a net decrease of *I_Ks_* [38].
- Uncommon LQTS mutations causing an increase in inward currents. Mutations in two different β-subunits regulators of the Na_V_1.5 channel have been found in families with LQTS: the β1 (encoded by the *SCN1B* gene) and the β4 (encoded by the *SCN4B* gene), inducing in both cases an increased *I_Na_* [39,40]. Increased *I_CaL_* currents are seen in LQTS-associated mutations on the *CACNA1C* gene encoding Ca_V_1.2, the α1C-subunit of the LTCC [41]. Specifically, mutations in this gene have also been associated with the Timothy syndrome, characterized by long QT and associated serious developmental and physical disorders such as autism or immune deficiencies [42]. This particular subtype (also known as LQT8) is considered the most severe variant of LQTS, with the highest mortality rate, generally caused by extracardiac complications [19]. Other genes encoding auxiliary proteins have been associated with LQTS. *ANK2* encodes ankyrin-2, a protein in charge of the assembly of the Na^+^/K^+^ exchanger, the Na^+^/Ca^2+^ exchanger and the inositol triphosphate receptor (IP3R), among others. Loss-of-function mutations in *ANK2* increase *I_CaL_* by decreasing the amount of Na^+^/Ca^2+^ exchanger in the membrane leading to an abnormal restoration of the initial ion state [43,44]. All three calmodulin-encoding genes (*CALM1*, *CALM2*, and *CALM3*) have also been linked to LQTS. Calmodulin is an essential intracellular Ca^2+^ sensor that acts as a signal-transducing protein and modulates Ca_V_1.2 (and others). Mutations in one of these genes, even in heterozygosis (meaning that only 1/6 alleles is affected), is sufficient to lead to an early and severe form of LQTS with extreme long QTc interval secondary to an impaired Ca_V_1.2 inactivation and increased *I_CaL_* currents [45,46,47,48,49]. Mutations in the *SNTA1* gene has also been associated to LQTS [50]. This gene encodes α1-syntrophin, a scaffolding protein that associates Na_V_1.5 channels with the nitric oxide synthase-ATPase plasma membrane Ca^2+^-transporting four-protein complex (NOS-PMCA4b). *SNTA1* mutations disrupt this interaction and increase late *I_Na_* currents [51,52]. Finally, mutations in the *TRDN* gene that encodes the triadin, a regulator of RyR, have been associated with the LQTS by putatively decreasing Ca_V_1.2 inactivation and increasing *I_CaL_* currents [18].

Other rare mutations have been related to the LQTS, with less established mechanisms. Mutations in *CAV3* may lead to LQTS by increasing *I_Na_* or by decreasing *I_K1_* currents. *CAV3* encodes caveolin-3 (Cav3), an anchoring protein that regulates membrane expression of multiple ion channels. Mutations in *CAV3* have been associated with loss of Kir2.1 (and a consequent decrease in *I_K1_*) [53] or a gain of Na_V_1.5 (increasing late *I_Na_* currents) [54], whereas other groups have found no association between these previously described *CAV3* mutations and LQTS [55]. A loss-of-function mutation in the *TRPM4* gene, encoding the transient receptor potential melastatin 4 (TRPM4), has been found in one family with LQTS history, with no established mechanism to explain such association [56]. Lastly, mutations in the *RYR2* gene encoding the RyR type 2 have also been reported in LQTS patients. Considering the strong implication of this gene in CPVT (see below) and the potential phenotypical overlap between both diseases, there is some controversy whether *RYR2* is a true LQTS-susceptible gene [57,58] or a CPVT gene putatively diagnosed as LQTS [59].

## 4. Short QT Syndrome

The short QT syndrome was first described in 2000 [60] and since then around 200 cases from 50 different families have been reported world-wide [8]. This highly-lethal rare disease is characterized by the presence of an abnormally short QTc interval on the ECG (Figure 2B) and a predisposition to develop life-threatening ventricular arrhythmias [60,61]. The short QT interval on the ECG reflects a short AP duration at the cellular level, caused by faster repolarization rates (and shorter refractory periods) that typically are heterogeneous along the cardiac tissue, favouring the occurrence of ventricular arrhythmias (Figure 3B) [6].

### 4.1. Clinical Features

The SQTS is diagnosed in the presence of a QTc ≤ 340 ms [13]. The diagnosis should also be considered in the presence of a QTc ≤ 360 ms together with the presence of a confirmed pathogenic mutation, a family history of SQTS or SCD at age <40, or a documented VT/VF episode [13]. Initial descriptions pointed to very aggressive clinical manifestations in SQTS patients, with up to 60% experiencing syncope and 31% SCD in follow-up, frequently at young age [62,63]. More recent publications highlight a great variability of phenotypes, with more than half patients remaining asymptomatic despite showing continuous short QTc on the ECG [64]. Other arrhythmias, such as atrial fibrillation, are common in SQTS patients [65].

Considering the low number of cases described to date, the optimal strategy for prevention of SCD is unclear [13]. Little is known about patient risk stratification [65]: neither the length of the QTc interval, the history of previous syncope, the gender or the genetic background have been shown to be predictors of the occurrence of arrhythmias [13]. Current guidelines recommend ICD implant in patients with SQTS who are survivors of a previous cardiac arrest and in those with documented sustained VT. Quinidine or sotalol (which prolong cardiac repolarization and therefore QT interval primarily due to inhibition of repolarizing currents) may be used in asymptomatic SQTS patients or in those with contraindications for ICD [10,13]. Given the limited yield of genetic testing in SQTS, recommendations in this regard are feeble [10,13].

### 4.2. Genetic Bases

Although SQTS is considered an inherited channelopathy whose clinical manifestations are linked to mutations in cardiac ion channels, only in 15% of cases is a responsible mutation found [8,65]. To date, six genes have been described in families with SQTS (Table 2). All associated mutations have been reported in genes exclusively encoding ion channel subunits and found to be inherited in an autosomal dominant pattern with high penetrance. The consequence of all SQTS-related mutations is a shortening of the cardiac AP that can be due either to an increase in repolarizing outward currents or a decrease in inward currents (Figure 3B).

*KCNH2* (associated with the so-called SQT1) seems to be the major SQTS-susceptible gene described so far [66]. Gain-of-function mutations in *KCNH2* encoding K_V_11.1 lead to an increase in *I_Kr_* by slowing the inactivation of the channel [67,68,69]. Gain-of-function mutations in the *KCNQ1* gene have also been related to SQTS, inducing an increase in *I_Ks_* either by accelerating the activation kinetics of K_V_7.1 [70] or by decreasing its inactivation due to an impaired regulation of minK, the β1-subunit of voltage-dependent K^+^ channels [71]. Finally, mutations in the *KCNJ2* gene have been linked to a shortening of the AP by shifting the voltage-dependency and increasing peak currents through Kir2.1 [72,73] or by enhancing the membrane expression of the channel [74].

Several loss-of-function mutations in genes encoding different subunits of the LTCC have been associated with the SQTS. Mutations in *CACNA1C*, encoding Ca_V_1.2, the α1C-subunit of the voltage-dependent LTCC, have been identified to shorten the AP by reducing the α1C-subunit traffic to the membrane [75]. On the other hand, a mutation in *CACNB2b*, encoding the β2-subunit of the LTCC, was shown to reduce dramatically *I_CaL_* without affecting traffic [75]. Both mutations in *CACNA1C* and *CACNB2b*, by decreasing inward currents at early phases of cell repolarization, also induce transmural and epicardial dispersion of repolarization, leading to a combined phenotype of BrS (with the characteristic ST elevation on the ECG) and SQTS (with short QTc interval) [75]. Finally, a mutation in *CACNA2D1* gene, encoding the α2δ1-subunit of LTCC, seemed to be related to a case of SQTS. Although the proposed mechanism was a decrease in *I_CaL_* currents through Ca_V_1.2 [76], genotype-positive relatives of the index case failed to display ECG features of SQTS, so at present it is not clear whether *CACNA2D1* is a real SQTS-causal gene or not [8].

## 5. Brugada Syndrome

First described in 1992, the BrS is characterized by a typical ECG pattern (coved-type ST-segment elevation in right precordial leads, Figure 2C) and a susceptibility to develop ventricular arrhythmias and SCD [77]. From experimental studies we now know that the characteristic ECG pattern responds to an imbalance between inward and outward currents in early phases of repolarization (phase 1 of the AP), created either by a decrease in inward (mainly *I_Na_*, and less importantly *I_CaL_*) or an increase in outward (*I_to_*, *I_Ks_*, or *I_Kr_*) currents (Figure 3C) [78]. This imbalance sets also the basis for the development of ventricular arrhythmias by a mechanism of phase 2 re-entry, initiated when voltages in phase 1 reach approximately −30 mV, potentially leading to a premature repolarization that could be affecting only a subset of cells, and not others. The heterogeneous repolarization across the cardiac tissue may facilitate then re-entrant arrhythmias [78]. However, the arrhythmogenic mechanism underlying the BrS has been and still is under extensive debate, with other authors considering the disease a primary depolarization disorder [79]. Taking into account the amount of genes associated with the BrS, with different outcomes in the AP (see below), one could speculate that the BrS is a heterogeneous disease potentially explained by more than one arrhythmogenic mechanism.

### 5.1. Clinical Features

The BrS is currently diagnosed in patients with a characteristic pattern of ST-segment elevation (defined as coved-type or type 1) ≥2 mm in ≥1 leads from V1 to V2 positioned in the second, third, or fourth intercostal space [13]. The ECG may be observed either spontaneously or after being unmasked by a provocative drug test with a sodium-channel blocker (Figure 2C) [13]. Sodium-channel blockers are antiarrhythmic agents that, by inhibiting *I_Na_*, increase the imbalance between inward and outward currents in early phases of the AP, and therefore may exacerbate the phenotypic expression of the BrS [80]. Of note, the BrS must be distinguished from other conditions, globally known as Brugada phenocopies, that may induce a Brugada-like ECG pattern in patients without the syndrome, among which metabolic and electrolyte disturbances, mechanical compression of the right ventricle, and acute ischemic or pericardial diseases are the most common [81].

The prevalence of the BrS is highly variable in different geographical areas, but it has been estimated in 5/10,000 inhabitants, although it could be higher given that many patients present concealed forms of the disease [78]. According to previous reports, the BrS could be responsible for 4–12% of all SCD and for up to 20% of SCD in subjects without structural heart disease [82]. Patients with BrS usually remain asymptomatic, but syncope or SCD due to ventricular arrhythmias have been described in 17–42% of diagnosed individuals [83,84]. Age at presentation is around the third-fourth decade of life [80]. For *SCN5A*-mutation carriers (the gene most commonly affected in BrS patients, see below), like in the case of LQT3 patients, symptoms typically appear during rest or sleep [80]. Gender differences have been reported, with the BrS being 8–10 times more prevalent in men, in whom the syndrome entails a worse prognosis [85]. Besides gender, other factors have been associated with an increased risk of life-threatening arrhythmias in patients with BrS. After extensive debate for more than a decade, referral authors have come to agree that a history of previous syncope, a spontaneous (not-induced) type-1 ECG and the inducibility of ventricular arrhythmias during programmed electrical stimulation (a catheter-based invasive test to test arrhythmia susceptibility) are all predictors of future SCD in BrS patients [84,86].

To date, ICD continues to be the only proven effective treatment to prevent SCD in BrS patients. Current guidelines recommend ICD implantation in all patients with previous arrhythmia-related symptoms [10,13]. ICD should also be considered in asymptomatic patients who develop ventricular arrhythmias during programmed ventricular stimulation [10,13]. Quinidine, a drug with blocking effects on the *I_to_* current (and thus the potential to decrease the ionic imbalance in phase 1 of the AP), has been proven useful in patients with contraindication for ICD, multiple ICD shocks, arrhythmic storms or in high-risk children [10,13,87]. Recently, catheter ablation of the underlying substrate in regions that are supposed to display the highest ionic imbalance (such as the epicardium of the right ventricular outflow tract) has become available in specialized centers and can be offered to high-risk patients with multiple arrhythmic episodes. Interestingly, this technique not only reduces the incidence of arrhythmias but also eliminates the ECG pattern in BrS patients [88].

### 5.2. Genetic Bases

The BrS is a genetically heterogeneous disease, with only 20–30% of all diagnosed patients having a known causal mutation and most associated genes having been described in anecdotic cases. This manifests a big gap of knowledge in the genetics of this disease [89]. Therefore, genetic testing is modestly recommended in BrS patients [10]. In general, all BrS-susceptible genes are inherited following an autosomal-dominant pattern, with the exception of *KCNE5*, which presents an X-linked inheritance. Nevertheless, considering the low disease penetrance observed in some families, some authors suggest that some cases of the syndrome could be associated with a much more complex pattern of inheritance [90].

To date, mutations in 25 different genes have been linked to the BrS, 18 of which encoding ion channel subunits and 7 encoding regulatory proteins (Table 3). With some exceptions, the two main molecular mechanisms that have been proposed to explain the BrS pattern are either a decrease in the *I_Na_* currents or an increase in the *I_to_* currents during early repolarization (Figure 3D). The major BrS-susceptible gene is *SCN5A* (BrS1), which encodes the α-subunit of the Na^+^ channel, Na_V_1.2, and is responsible of 25% of all genetically-diagnosed patients [91]. More than 300 different mutations in *SCN5A* have been associated with the disease, most leading to a loss of function either by impairing traffic or by modifying the channels’ gating properties [92]. The exact molecular mechanism depends on the location of the mutation within the channel, which can also determine the clinical severity of the disease [93]. Two different groups have demonstrated that, among BrS patients, those with mutations leading to a truncated protein, presumably causing greater *I_Na_* decrease, have more severe phenotypes [93,94]. Mutations affecting any other regulatory subunit of the Na^+^ channel are also candidates for BrS. Indeed, mutations in genes encoding 3 β-subunits that regulate Na_V_1.2 traffic and function (*SCN1B*, *SCN2B* and *SCN3B*) and consequently reduce *I_Na_* currents have been described in BrS patients [95,96,97,98]. The *SCN10A* gene, encoding Na_V_1.8, the neuronal Na^+^ channel also present in the intracardiac terminal neurons, has also been linked to the BrS [99]. Some authors have proposed this as a major-susceptibility gene, given that in some series *SCN10A* mutations have been found in up to 10% of all BrS patients [100]. However other authors have failed to reproduce these findings [101].

Reduction of inward currents other than *I_Na_* could potentially induce the Brugada pattern, by creating a similar imbalance in early phases of repolarization. As such, reduced *I_CaL_* currents, secondary to loss-of-function mutations affecting Ca_V_1.2 or the regulatory subunits β2 and α2δ1 (encoded by *CACNA1C*, *CACNB2b* and *CACNA2D1* respectively), have also been reported as causative of BrS [75,102,103]. Interestingly, because *I_CaL_* also contributes to later phases of repolarization, mutations in these genes have also been associated with a reduction in the AP duration and a short QT interval on the ECG, leading to a combined phenotype of BrS and SQTS [75,102,103].

Mutations in several subunits that mediate the *I_to_* currents have also been associated with the BrS. Gain-of-function mutations in *KCND3*, which encodes K_V_4.3, the α-subunit of the voltage-dependent K^+^ channel mediator of the transient *I_to_* current, induce BrS by direct increase in *I_to_* currents [104]. Similarly, gain-of-function mutations in the *KCNE3* gene, encoding the minK-related peptide 2, the β-subunit that interacts with K_V_4.3 and regulates its current density, lead also to BrS [105,106,107]. Other sporadic mutations indirectly affecting *I_to_* have also been described in BrS patients. A mutation in the *KCNAB2* gene encoding the β2-subunit that regulates K_V_4.3 traffic to the membrane was found to increase *I_to_* currents by enhancing the membrane expression of the channel and induce BrS [108]. Mutations in *KCND2*, which encodes K_V_4.2, another contributor to the *I_to_* current, have also been found in BrS patients, who show a 50%-increased peak in *I_to_* [109]. Mutations in the *KCNE5* gene, encoding the minK-related peptide 4, a β-subunit that regulates K_V_7.1, increase currents through K_V_4.3 without affecting K_V_7.1, suggesting that minK-related peptide 4 is also a modulator of the *I_to_* current and a putative BrS-susceptible gene [110].

By a mechanism similar to that proposed for mutations causing an increase in *I_to_* currents, mutations inducing increases in other repolarizing currents may predispose to BrS. Increases in the *I_K-ATP_* currents have been seen in gain-of-function mutations of *KCNJ8* and *ABCC9*, encoding respectively Kir6.1 and its modulator SUR2, and explain some BrS cases [111,112,113]. Similarly, several mutations in *KCNH2* that affect the N or C-terminal tails of K_V_11.1, the hERG channel, have shown to result in increased *I_Kr_* and potentially induce BrS [68,114,115].

Less is known about the mechanisms of the mutations found in other genes encoding ion channels that have been found in BrS patients. Loss-of-function mutations in *HCN4* gene, which encodes the pacemaker channel (hyperpolarization-activated, cyclic nucleotide-gated ion channel 4), mediator of the *I_f_* current and responsible for the spontaneous activity of the sinoatrial node (SAN), have been reported in two different works [116,117]. Finally, mutations in the TRPM4 channel, which, like HCN4, seems to participate in the diastolic depolarization that gives rise to the AP in the SAN, have been found in BrS patients and proposed as related to the conduction disorders that may appear in the disease [118,119]. Strikingly, both gain-of-function and loss-of-function *TRPM4* mutations have been described in BrS patients, reflecting that much more investigation needs to be done in this regard [120].

Among the seven genes that have been associated with the BrS that encode regulatory proteins and not ion channels, five of them interact with *I_Na_* currents. *FGF12* gene encodes the fibroblast growth homologous factor 12, a potent regulator of Na_V_1.5 traffic and function [121]. Mutations in this gene associated with BrS were found to strongly reduce Na^+^ but not Ca^2+^ currents [122]. Severe traffic defects of Na_V_1.5 with the consequent reduction in *I_Na_* currents have also been reported in patients with mutations in the *GPD1L* gene, encoding the glycerol-3-phosphate dehydrogenase 1-like protein, and the *SLMAP* gene, encoding the sarcolemma associated protein [123,124,125]. A dominant negative mutant of MOG1 (encoded by the *RANGRF* gene) was described to also decrease Na_V_1.5 traffic to the membrane in BrS patients [126]. Nevertheless, the same mutation was found in non-affected relatives and complete knock-down of MOG-1 was tolerated without any sign of disease, questioning whether this is a true BrS-susceptible gene [127,128]. Moreover, mutations that affect the expression of the desmosomal protein plakophillin-2 (encoded by *PKP2*) also have been shown to reduce the number of Na_V_1.5 channels at the intercalated disc, possibly explaining the molecular substrate for BrS in these patients [129]. The last two regulatory proteins associated with BrS interact with *I_to_* currents. *SEMA3A* gene encodes for semaphorin-3A, a protein that binds to K_V_4.3 and reduces peak current densities without perturbing cell surface expression. In the context of BrS, loss-of-function mutations of *SEMA3A* lead to an increase in *I_to_* currents [130]. Finally, a recent genome-wide association study (GWAS) identified a strong association between a region near the *HEY2* gene and BrS [131]. This gene encodes a transcription factor that regulates electric patterning across the ventricular wall and affects cardiac ion channel gene expression. Although no BrS-associated mutation has been described so far, genome-wide co-expression analysis postulated KCNIP2, the β-subunit regulator of the *I_to_* current, as one of the regulated genes by *HEY2* and potentially associated with BrS [132].

## 6. Catecholaminergic Polymorphic Ventricular Tachycardia

Although the earliest clinical reference goes back to 1975 [133], the first series of catecholamine-induced tachycardia or CPVT was not described until 20 years later [134]. CPVT, although not a pure channelopathy, was included among the primary electrical disorders much later [59]. This rare but extremely severe disease is characterized by the presence of a normal ECG at baseline but the predisposition to adrenergic-induced ventricular tachycardias that typically are bidirectional or polymorphic (Figure 2D) [135] and can induce SCD. Mechanistic studies have elucidated the molecular basis of CPVT, which relies on an abnormal release of Ca^2+^ from the SR in response to adrenergic stimulation. Excess Ca^2+^ is handled by the cell membrane Na^+^/Ca^2+^-exchanger, which transports three Na^+^ ions into the cell per single Ca^2+^ ion extruded, creating a net depolarizing current that can lead to arrhythmogenesis by a mechanism called delayed afterdepolarizations (Figure 3D) [136,137].

### 6.1. Clinical Features

According to the 2015 European Society of Cardiology guidelines, CPVT can be currently diagnosed (class I recommendation): (1) in the presence of a structurally normal heart, normal ECG, and exercise- or emotion-induced bidirectional or polymorphic ventricular tachycardia; (2) in patients who are carriers of a pathogenic mutation in *RYR2* or *CASQ2* genes [13]. However, it is important to note that mutations in other genes have recently been identified in patients with clinical features of CPVT (see below). With an estimated prevalence of 1 in 10,000 [138], CPVT is an inherited disorder with both autosomal dominant and recessive patterns of transmission. Although an incomplete penetrance has been reported (around 15% of all patients are silent carriers), CPVT is usually an aggressive disorder, with symptoms likely appearing during childhood and a high incidence of cardiac events in follow-up (around 80% of untreated patients will experiment an arrhythmia, and up to 30% SCD) [139]. Due to the particular mechanism of this condition, arrhythmia-related symptoms such as syncope typically occur in adrenergically mediated circumstances such as exercise or emotional stress [135]. Male-sex seems to be a risk factor in patients carrying mutations in *RYR2* [135]. No other predictors of risk have been identified to date [13].

Besides avoidance of competitive sports or strenuous exercise and stressful environments, all CPVT patients should receive treatment with β-blockers upon diagnosis to prevent SCD [13]. However, up to 45% of patients may still experience symptoms under treatment. In these cases, an ICD is recommended although additional flecainide may be useful [10,13,140,141,142]. There is still some debate regarding the mechanism by which flecainide reduces the incidence of arrhythmias, with some authors postulating that flecainide modulates RyR2 thus reducing Ca^2+^ leak, and some others supporting that it blocks Na_V_1.2, raising the threshold for delayed afterdepolarizations [143,144]. For survivors of a previous cardiac arrest, an ICD is warranted [13]. Genetic testing in CPVT might be reasonable, although its diagnostic yield is modest, with approximately 60% having a recognized causative mutation [10].

### 6.2. Genetic Bases

CPVT is the only arrhythmogenic disorder that does not affect directly the cardiac AP. As mentioned before, the molecular mechanism leading to delayed depolarizations and arrhythmias is an impaired regulation of the SR Ca^2+^ handling. All the described CPVT-related genes thus far affect in one way or another RyR2, the channel responsible of Ca^2+^ release from the SR.

Although seven genes have been associated with CPVT (Table 4), 60% of all patients carry a mutation in *RYR2* gene [145]. Mutations in this protein, inherited in an autosomal dominant pattern, concentrate in three regions: surrounding the Ca^2+^-binding site, in the transmembrane segments and in the FKBP12.6-binding domain, a protein that regulates RyR2 gating [146]. In all cases, *RYR2* mutations impair normal RyR2 functioning, causing opening and Ca^2+^ leak during diastole [137]. Similarly, mutations in the *CASQ2* gene, present in 5% of all genetically-diagnosed CPVT patients, also lead to an abnormal Ca^2+^ leak through RyR2 by decreasing the expression of its encoded protein, calsequestrin 2 [147]. Calsequestrin 2 is the main Ca^2+^-buffering protein in the SR. If its expression is reduced, free-Ca^2+^ in the SR increases leading to a RyR2 leak [148]. These mutations, inherited in a dominant or recessive manner, are associated to a higher rate of sudden death [149,150,151]. The physical link between RyR2 and calsequestrin 2 is found in triadin, a protein encoded by *TRDN* [152]. Mutations in this protein, recessively inherited, result in its complete degradation and SR leak due to impaired regulation of the complex calsequestrin-triadin-RyR2 [153].

Additionally, mutations in the three calmodulin-encoding genes have also been associated to CPVT. In this context, CaM is a regulator of Ca^2+^ handling that avoids Ca^2+^ leak during diastole. *CALM1* mutations disrupt the interaction between CaM and the RyR2 favoring the Ca^2+^ leak [154]. *CALM2* and *CALM3* mutations do not disrupt the interaction but lower the CaM-Ca^2+^-binding affinity, prompting spontaneous Ca^2+^ waves and spark activity, phenotype that mimics an increase in RyR2 function [46,155]. Nevertheless, mutations in *CALM2* have been found in patients with a double diagnosis for LQTS and CPVT, and considering the overlapping features of both diseases, one should be cautious in establishing *CALM2* as a unique CPVT-susceptible gene.

Recently, the TECLR gene was also identified as a CPVT-susceptible gene. Recessively-inherited mutations in the gene encoding for the trans-2,3-enoyl-CoA reductase-like protein were found in three different non-related families [156]. This protein, strongly expressed in the heart and localized in the SR, is thought to participate in the synthesis of fatty acids. Mutations in this gene correlated with a reduction in RyR2 and calsequestrin-2 protein levels and a consequent impairment in calcium handling

Mutations in *ANK2* and *KCNJ2* genes have been reported in patients with exercise-induced bi-directional ventricular tachycardia, thus mimicking the clinical phenotype of CPVT. However, it is now believed that these mutations, previously linked to the LQT4 and LQT7-Andersen–Tawil syndrome, respectively, are clinical phenocopies of CPVT (Priori ESC guidelines) and should be included in the differential diagnosis of this entity. In the case of *ANK2* mutations, the clinical phenotype might be characterized by a normal ECG at rest and polymorphic ventricular tachycardias induced by exercise, with an overall better outcome than with the CPVT [157]. These disorders have recently been proposed as a distinct entity known as the Ankyrin B syndrome [158]. *KCNJ2* mutations responsible for the Andersen–Tawil syndrome typically manifest with the phenotypic triad of ventricular arrhythmias, commonly induced by exercise, periodic paralysis, and facial and limb dysmorphism, but these latter features might be variably present, which, together with a mild QTc prolongation, may very much mimic CPVT [159]. However, distinction with true CPVT is important because patients with Andersen–Tawil syndrome show a much more benign course [35,159].

## 7. Conclusions

The normal electric function of the heart is exquisitely orchestrated by macromolecular complexes with ion channels in its core. These complexes include proteins regulating functions from transcription to function or degradation of such channels. Any disruption of this gear assembly may generate electrical instability and a substrate capable of inducing ventricular arrhythmias and SCD [160]. Therefore, it is extremely important to gain knowledge on the electrophysiological mechanisms that control ion channel functions. At present, there are still many unanswered questions, such as why mutations in the same gene and with the same general output can cause different arrhythmogenic disorders, or why patients carrying the same mutation may present a diverse phenotype, from remaining asymptomatic throughout life to experiencing SCD at an early age. The most probable explanation to this puzzle is that, apart from the identified mutations, we are missing modifying factors that modulate the outcome in a mutation- and patient-specific manner. Understanding these diverse mechanisms may guide us to design directed therapies for inherited arrhythmogenic syndromes in the future.

## Figures and Tables

**Table 1:** Mutations associated with the LQTS.

Gene	Protein	Current	Effect	Function	Prevalence
GENES ENCODING ION CHANNEL SUBUNITS					
1. Major LQTS-susceptibility genes					
*KCNQ1*	K_V_7.1 (α-subunit of the voltage-dependent K^+^ channel)	↓ *I_Ks_*	loss-of-function	mediator of the slow component of the delayed rectifying potassium *I_Ks_* current	⋍40% (LQT1)
*KCNH2*	K_V_11.1/hERG (α-subunit of the voltage-dependent K^+^ channel)	↓ *I_Kr_*	loss-of-function	mediator of the rapid component of the delayed rectifying potassium *I_Kr_* current	⋍30% (LQT2)
*SCN5A*	Na_V_1.5 (α-subunit of the voltage-dependent Na^+^ channel)	↑ *I_Na_*	gain-of-function	mediator of the depolarizing inward sodium *I_Na_* current	⋍10% (LQT3)
**2. Rare LQTS-susceptibility genes**					
**By reducing outward currents**					
*KCNE1*	minK (β1-subunit of the voltage-dependent K^+^ channel)	↓ *I_Ks_*	loss-of-function	auxiliary protein modulator of K_V_7.1 and the *I_Ks_* current	<1%
*KCNE2*	MiRP1 (β2-subunit of the voltage-dependent K^+^ channel)	↓ *I_Kr_*	loss-of-function	auxiliary protein modulator of K_V_11.1 and the *I_Kr_* current	<1%
*KCNJ2*	Kir2.1 (inward rectifying K^+^ channel)	↓ *I_K1_*	loss-of-function, extra-cardiac manifestations	mediator of the inward rectifying potassium *I_K1_* current	<1% (Andersen-Tawil syndrome, LQT7)
*KCNJ5*	Kir3.4 (G protein-activated inward rectifying K^+^ channel 4)	↓ *I_K,Ach_*	loss-of-function	mediator of the acetylcholine/adenosine-induced potassium *I_K,Ach_* current	<1%
**By increasing inward currents**					
*SCN1B*	β1-subunit of the voltage-dependent Na^+^ channel	↑ *I_Na_*	gain-of-function	auxiliary protein modulator of Na_V_1.5 and the *I_Na_* current	<1%
*SCN4B*	β4-subunit of the voltage-dependent Na^+^ channel	↑ *I_Na_*	gain-of-function	auxiliary protein modulator of Na_V_1.5 and the *I_Na_* current	<1%
*CACNA1C*	Ca_V_1.2 (α1C-subunit of the voltage-dependent L-type Ca^2+^ channel)	↑ *I_CaL_*	gain-of-function, extra-cardiac manifestations	mediator of the inward calcium *I_CaL_* current	<1% (Timothy syndrome, LQT8)
**GENES ENCODING AUXILIARY PROTEINS**					
**By reducing outward currents**					
*AKAP9*	A-kinase anchor protein-9	↓ *I_Ks_*	disruption of K_V_7.1/PKA interaction	scaffolding protein assembling PKA and K_V_7.1	<1%
**By increasing inward currents**					
*ANK2*	ankyrin B	↑ *I_CaL_*	disruption of Na^+^/K^+^ exchanger, Na^+^/Ca^2+^ exchanger/IP_3_ interaction	scaffolding protein assembling Na^+^/K^+^ exchanger, Na^+^/Ca^2+^ exchanger and IP_3_ receptor	<1%
*CALM1*	calmodulin (CaM)	↑ *I_CaL_*	disorder in Ca_V_1.2 functioning	essential Ca^2+^ sensor, signal-transducing protein modulator of Ca_V_1.2 (and others)	<1%
*CALM2*	calmodulin (CaM)	↑ *I_CaL_*	disorder in Ca_V_1.2 functioning	essential Ca^2+^ sensor, signal-transducing protein modulator of Ca_V_1.2 (and others)	<1%
*CALM3*	calmodulin (CaM)	↑ *I_CaL_*	disorder in Ca_V_1.2 functioning	essential Ca^2+^ sensor, signal-transducing protein modulator of Ca_V_1.2 (and others)	<1%
*SNTA1*	α1-syntrophin	↑ *I_Na_*	disruption of Na_V_1.5/NOS-PMCA4b complex interaction	scaffolding protein that associates Na_V_1.5 channels with the NOS-PMCA4b complex	<1%
*TRDN*	triadin	↑ *I_CaL_*	reduction of *I_CaL_* inactivation	regulator of ryanodine receptors and Ca_V_1.2	<1%
**Less established mechanisms**					
*CAV3*	caveolin-3	↑ *I_Na_*?/↓ *I_K1_*?	changes in membrane expression of Na_V_1.5/Kir2.1	scaffolding protein regulating ion channels in caveolae	<1%
*TRPM4*	Transient receptor potential melastatin 4		loss-of-function	regulator of conduction and cellular electrical activity which impact heart development	<1%
*RYR2*	ryanodine receptor 2 (RyR2)		*not described*	mediator of Ca^2+^ release from the SR	<1%

↑: increased current; ↓: decreased current; ?: suspected but not confirmed mechanism.

**Table 2:** Mutations associated with the SQTS.

Gene	Protein	Current	Effect	Function	Prevalence
GENES ENCODING ION CHANNEL SUBUNITS					
By increasing outward currents					
*KCNH2*	K_V_11.1/hERG (α-subunit of the voltage-dependent K^+^ channel)	↑ *I_Kr_*	gain-of-function	mediator of the rapid component of the delayed rectifying potassium *I_Kr_* current	⋍15% (SQT1)
*KCNQ1*	K_V_7.1 (α-subunit of the voltage-dependent K^+^ channel)	↑ *I_Ks_*	gain-of-function	mediator of the slow component of the delayed rectifying potassium *I_Ks_* current	<1%
*KCNJ2*	Kir2.1 (inward rectifying K^+^ channel)	↑ *I_K1_*	gain-of-function	mediator of the inward rectifying potassium *I_K1_* current	<1%
**By decreasing inward currents**					
*CACNA1C*	Ca_V_1.2 (α1C-subunit of the voltage-dependent L-type Ca^2+^ channel)	↓ *I_CaL_*	loss-of-function, combined phenotype of SQTS and BrS	mediator of the inward calcium *I_CaL_* current	<1%
*CACNB2b*	β2-subunit of the voltage-dependent L-type Ca^2+^ channel	↓ *I_CaL_*	loss-of-function, combined phenotype of SQTS and BrS	auxiliary protein modulator of Ca_V_1.2 and the *I_CaL_* current	<1%
**Less established mechanisms**					
*CACNA2D1*	α2/δ-subunit of the voltage-dependent L-type Ca^2+^ channel	↓ *I_CaL_?*	loss-of-function?	auxiliary protein modulator of Ca_V_1.2 and the *I_CaL_* current	<1%

↑: increased current; ↓: decreased current; ?: suspected but not confirmed mechanism.

**Table 3:** Mutations associated with the BrS.

Gene	Protein	Current	Effect	Function	Prevalence
GENES ENCODING ION CHANNEL SUBUNITS					
1. Major BrS-susceptibility genes					
*SCN5A*	Na_V_1.5 (α-subunit of the voltage-dependent Na^+^ channel)	↓ *I_Na_*	loss-of-function	mediator of the depolarizing inward sodium *I_Na_* current	⋍25% (BrS1)
**2. Rare BrS-susceptibility genes**					
**By decreasing inward currents**					
*SCN1B*	β1-subunit of the voltage-dependent Na^+^ channel	↓ *I_Na_*	loss-of-function	auxiliary protein modulator of Na_V_1.5 and the *I_Na_* current	<1%
*SCN2B*	β2-subunit of the voltage-dependent Na^+^ channel	↓ *I_Na_*	loss-of-function	auxiliary protein modulator of Na_V_1.5 and the *I_Na_* current	<1%
*SCN3B*	β3-subunit of the voltage-dependent Na^+^ channel	↓ *I_Na_*	loss-of-function	auxiliary protein modulator of Na_V_1.5 and the *I_Na_* current	<1%
*SCN10A*	Na_V_1.8 (α-subunit of the neuronal voltage-dependent Na^+^ channel)	↓ *I_Na_*	loss-of-function	mediator of the depolarizing phase of the neural AP, associated with pain perception	⋍10%?
*CACNA1C*	Ca_V_1.2 (α1C-subunit of the volatge-dependent L-type Ca^2+^ channel)	↓ *I_CaL_*	loss-of-function, combined phenotype of BrS and SQTS	mediator of the inward calcium *I_CaL_* current	<1%
*CACNB2b*	β2-subunit of the voltage-dependent L-type Ca^2+^ channel	↓ *I_CaL_*	loss-of-function, combined phenotype of BrS and SQTS	auxiliary protein modulator of Ca_V_1.2 and the *I_CaL_* current	<1%
**By increasing outward currents**					
*KCND3*	K_V_4.3 (α-subunit of the voltage-dependent K^+^ channel)	↑ *I_to_*	gain-of-function	mediator of the transient outward K^+^ *I_to_* current	<1%
*KCNE3*	minK-related peptide 2 (β-subunit of the voltage-dependent K^+^ channel)	↑ *I_to_*	gain-of-function	regulator of K_V_4.3	<1%
*KCNAB2*	β2-subunit of the voltage-dependent K^+^ channel	↑ *I_to_*	gain-of-function	interaction with K_V_4.3	<1%
*KCND2*	K_V_4.2 (voltage-dependent K^+^ channel)	↑ *I_to_*	gain-of-function	contributor to the transient outward K^+^ *I_to_* current	<1%
*KCNE5*	minK-related peptide 4 (β-subunit of the voltage-dependent K^+^ channel)	↑ *I_to_*	gain-of-function	inhibitor of the delayed rectifying K_V_7.1 channel and modulator of K_V_4.3	<1%
*KCNJ8*	Kir6.1 (inward-rectifier K^+^ channel, subunit of the ATP-sensitive K^+^ channel)	↑ *I_K-ATP_*	gain-of-function	mediator of the *I_K-ATP_* currents	<1%
*ABCC9*	SUR2 (sulfonylurea receptor, subunit of the ATP-sensitive K^+^ channel)	↑ *I_K-ATP_*	gain-of-function	modulator of *I_K-ATP_* currents	<1%
*KCNH2*	K_V_11.1/hERG (α-subunit of the voltage-dependent K^+^ channel)	↑ *I_Kr_*	gain-of-function	mediator of the rapid component of the delayed rectifying potassium *I_Kr_* current	<1%
**Less established mechanisms**					
*CACNA2D1*	α2/δ subunit of the volatge-dependent L-type Ca^2+^ channel	↓ *I_CaL_?*	loss-of-function?, combined phenotype of SQTS and BrS	auxiliary protein modulator of Ca_V_1.2 and the *I_CaL_* current	<1%
*HCN4*	hyperpolarization-activated, cyclic nucleotide-gated ion channel 4	↓ *I_f_?*	loss-of-function?	mediator of the pacemaker current, *I_f_*	<1%
*TRPM4*	Transient receptor potential melastatin 4		loss-of-function/gain-of-function	regulator of conduction and cellular electrical activity which impact heart development	<1%
**GENES ENCONDING AUXILIARY PROTEINS**					
*FGF12*	fibroblast growth factor 12	↓ *I_Na_*	interaction with Na_V_1.5 trafficking	modulator of Nav1.5 and the *I_Na_* current	<1%
*GPD1L*	glycerol-3-phosphate dehydrogenase 1-like	↓ *I_Na_*	interaction with Na_V_1.5 trafficking	modulator of Na1.5 and the *I_Na_* current	<1%
*SLMAP*	sarcolemma associated protein (striatin-interacting phosphatase and kinase complex)	↓ *I_Na_*	interaction with Na_V_1.5 trafficking	present in the T-tubules, regulator of excitation-contraction coupling	<1%
*PKP2*	plakophillin-2	↓ *I_Na_*	changes in Na_V_1.5 expression in intercalated disc	binds to and modulates Na_V_1.5 and the *I_Na_* current	<1%
*SEMA3A*	semaphorin-3A	↑ *I_to_*	loss-of-function	inhibitor of the K_V_4.3 channel	<1%
**Less established mechanisms**					
*RANGRF*	MOG1 (multicopy suppressor of *Gsp1*)	↓ *I_Na_*?	interaction with Na_V_1.5 trafficking	involved in nuclear protein import—regulates cell surface location of Na_V_1.5	<1%
*HEY2*	CHF1 (cardiovascular helix-loop-helix factor 1)	↑ *I_to_*?	interaction with KCNIP2	transcriptional regulator of cardiac electrical function	<1%

↑: increased current; ↓: decreased current; ?: suspected but not confirmed mechanism.

**Table 4:** Mutations associated with the CPVT.

Gene	Protein	Effect	Function	Prevalence
GENES ENCODING ION CHANNELS AND AUXILIARY PROTEINS				
1. Major CPVT-susceptibility genes				
*RYR2*	ryanodine receptor 2 (RyR2)	cytoplasmic Ca^2+^ overload, due to Ca^2+^ leak from the SR	mediator of the release of stored Ca^2+^ ions from the SR	⋍50–60% (CPVT1)
*CASQ2*	calsequestrin 2	decreased Ca^2+^ content in the SR and abnormal Ca^2+^ regulation	Ca^2+^ storage protein, controls Ca^2+^ release from the SR	⋍5%
**2. Rare CPVT-susceptibility genes**				
*TRDN*	triadin	cytoplasmic Ca^2+^ overload, due to Ca^2+^ leak from the SR	regulator of ryanodine receptors, controls the Ca^2+^ release from the SR	<1%
*CALM1*	calmodulin (CaM)	Ca^2+^ leak from the SR due to loss of interaction CaM-RyR2	essential Ca^2+^ sensor, signal-transducing protein modulator of Ca_V_1.2 or RyR2 (and others)	<1%
*CALM2*	calmodulin (CaM)	reduction in Ca^2+^-binding affinity in the CaM C-domain	essential Ca^2+^ sensor, signal-transducing protein modulator of Ca_V_1.2 or RyR2 (and others)	<1%
*CALM3*	calmodulin (CaM)	reduction in Ca^2+^-binding affinity in the CaM C-domain and leak from the SR	essential Ca^2+^ sensor, signal-transducing protein modulator of Ca_V_1.2 or RyR2 (and others)	<1%
*TECLR*	trans-2,3-enoyl-CoA reductase- like	decreased Ca^2+^ content in the SR and abnormal Ca^2+^ regulation	participates in the synthesis of fatty acids	<1%
